# Successful treatment with pazopanib plus PD-1 inhibitor and RAK cells for advanced primary hepatic angiosarcoma: a case report

**DOI:** 10.1186/s12885-018-3996-3

**Published:** 2018-02-21

**Authors:** Yu Qiao, Jihong Yang, Lili Liu, Yixin Zeng, Jie Ma, Jing Jia, Li Zhang, Xiaoguang Li, Peihong Wu, Wenchao Wang, Dongge Liu, Huan Chen, Yunbo Zhao, Huan Xi, Yao Wang

**Affiliations:** 1 Department of Geriatric, Beijing Hospital, National Center of Gerontology, Beijing, 100730 People’s Republic of China; 2 Department of Oncology, Beijing Hospital, National Center of Gerontology, Beijing, 100730 People’s Republic of China; 3 Department of Nephrology, Beijing Hospital, National Center of Gerontology, Beijing, 100730 People’s Republic of China; 4 Biological Treatment Center, Beijing Hospital, National Center of Gerontology, Beijing, 100730 People’s Republic of China; 5 Departmnet of Oncology, Tumor Hospital, Zhongshan University, Guangzhou, 510089 People’s Republic of China; 6 Minimally Invasive Tumor Therapies Center, Beijing Hospital, National Center of Gerontology, Beijing, 100730 People’s Republic of China; 7 The Center of Medical Image Guided Minimally Invasive Therapy, Tumor Hospital, Zhongshan University, Guangzhou, 510089 People’s Republic of China; 8 Department of Imaging, Beijing Hospital, National Center of Gerontology, Beijing, 100730 People’s Republic of China; 9 Department of Pathology, Beijing Hospital, National Center of Gerontology, Beijing, 100730 People’s Republic of China

**Keywords:** Primary hepatic angiosarcoma, Pazopanib, PD-1 inhibitor, RAK cell

## Abstract

**Background:**

Primary hepatic angiosarcoma (PHA) is a rare and aggressive solid tumor, with high rates of local recurrence and distant metastasis, and poor prognosis. There are no established treatment guidelines for PHA.

**Case presentation:**

A 78-year-old asymptomatic man with PHA that was successfully treated with pazopanib plus PD-1 inhibitor and RetroNectin-activated killer cells (RAK cells). After one month of treatment, there was a clear reduction in the size and number of the liver metastases; and after nearly 15 months, most of the lesions were stable, no new lesions had developed, and the side effect of treatment was minor.

**Conclusion:**

Pazopanib, PD-1 inhibitor and RAK cells could serve as a potential option for the treatment of advanced PHA.

## Background

Primary hepatic angiosarcoma (PHA) is a rare and aggressive solid tumor, with high rates of local recurrence and distant metastasis, and poor prognosis. Histopathology shows a variety of patterns of vascular channels, dilated sinusoidal or cavernous spaces, CD34, CD31 and factor VIII-related antigen can be positive [1]. Most effective treatment modality is liver resection, however, there are no established chemotherapy regimens and moreover, chemotherapy is only palliative [2].

## Case presentation

**Fig. 1:**
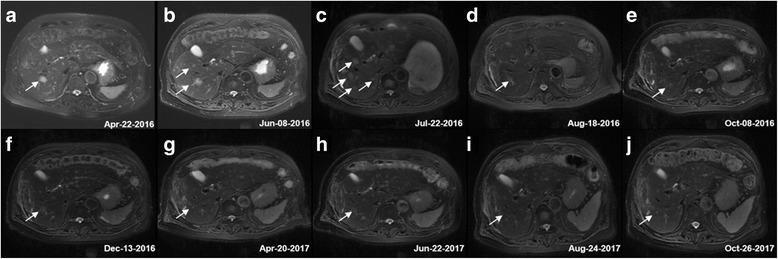
Abdomen MRI T2WI changes in liver lesions. **a** Apr-22-2016 MRI showing multiple hepatic lesions found. **b** Jun-08-2016 MRI showing hepatic lesions increase in number and size. **c** Jul-22-2016 MRI showing new lesions emerge after RFA. **d** Aug-18-2016 MRI showing tumor up to PR after first cycle of pazopanib plus PD-1 inhibitor and RAK cell. **e**-**j** From Oct-08-2016 to Oct-26-2017 MRI showing tumor stable disease. Arrows indicate the lesion. MRI, magnetic resonance imaging; PR, partial response; RAK cell. RetroNectin-activated killer cells

**Fig. 2:**
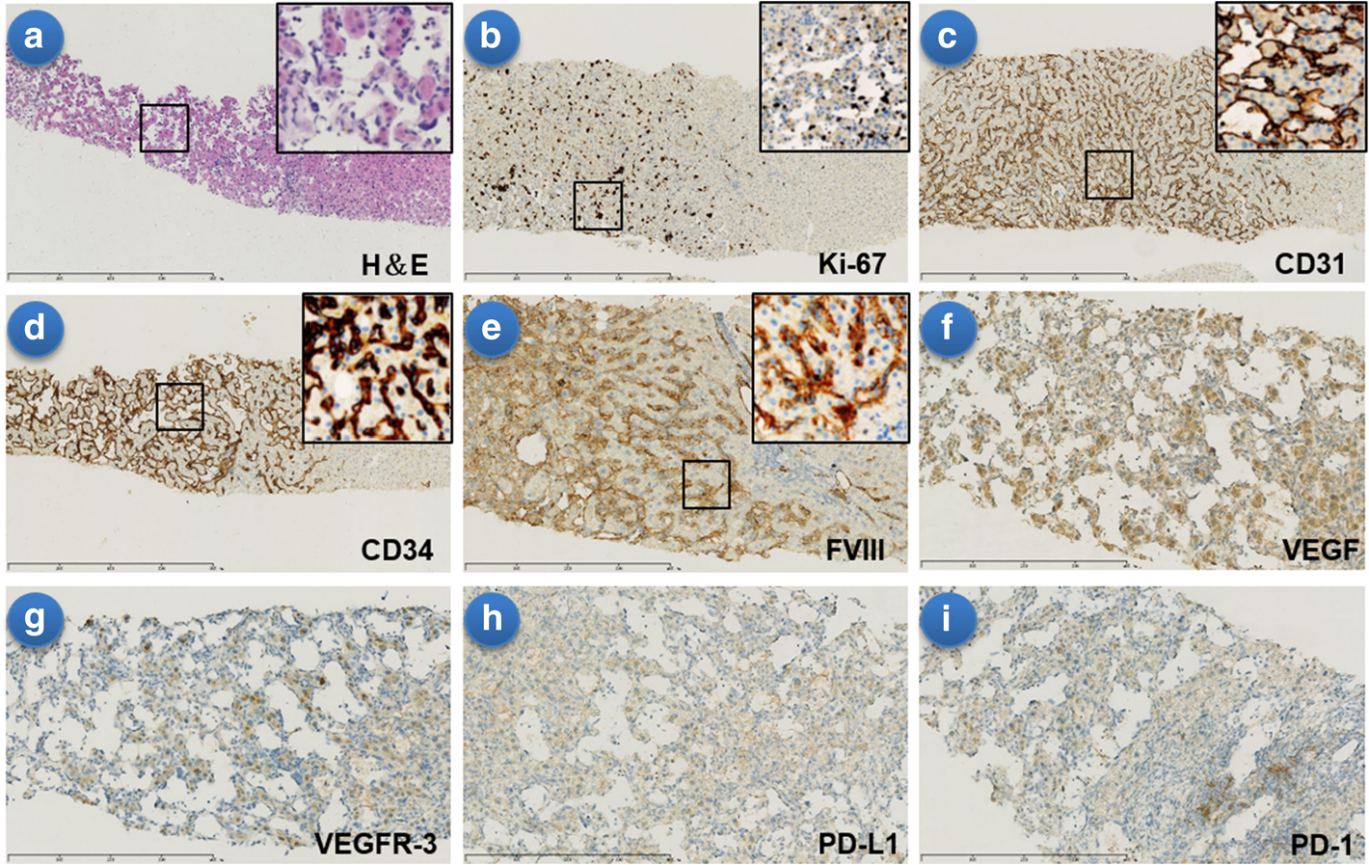
Histopathology staining of the core needle liver biopsy specimen. **a** H&E low-magnification view (× 40) and high-magnification view (× 400) in black frame showing sinusoids lined by atypical endothelial cells with marked nuclear pleomorphism, and vascular channels. **b**-**f** Immunohistochemistry low-magnification view (× 40–100) and high-magnification view (× 200–400) in black frame showing the cells were positive for Ki-67, CD31, CD34, FVIII and VEGF respectively. **g-i** Immunohistochemistry magnification view (× 100–200) showing the cells were negative for VEGFR3, PD-1 and PD-L1 respectively. H&E, hematoxylin and eosin

**Fig. 3:**
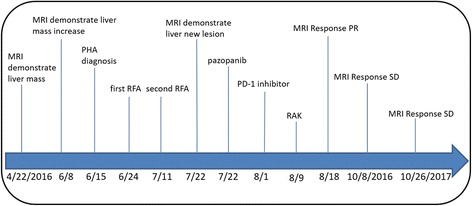
Timeline of patient’s clinical course

A 78-year-old asymptomatic man, who was found to have multiple liver masses by magnetic resonance imaging (MRI) (Fig. 1a) done as part of his routine medical examination on Apr 22,2016. These masses increased in size over six weeks (Fig. 1b), with no symptoms or abnormal findings on his physical examination. There is no treatment during this time. He was admitted in our hospital on June 13,2016. Liver function tests, hematology parameters as well as tumor markers such as α-fetoprotein (AFP), carbohydrate antigen 199 (CA199), Carcinoembryonic antigen (CEA), and chromogranin A (CgA) were all normal. Percutaneous liver biopsy was performed in two days later, pathology revealed hepatic endothelial cells predominately proliferating in the dilated sinusoid, atypical endothelial cells with marked nuclear pleomorphism. The immunochemistry showed CD34(+++), CD31(+++), FVIII(+), Ki-67(50%+), CD3(−), CD20(−), CD68(−), CD163(−), GPC3(−), HCC(−), CD5(−), CK19(−), PD-1(−), PD-L1(−), C-MET(−), ROS-1(−), VEGF(+), EGFR(−), HER2(−), ALK D5F3(−), VEGFR2(60%+), VEGFR3(−). (Fig. 2a–i). Final pathologic diagnosis was primary hepatic angiosarcoma. Endoscopy was normal. Positron emission tomography/computed tomography (PET/CT) showed no metastatic lesions. Next generation sequencing (NGS) using the TruSeq Amplicon-Cancer 295 gene panel (Guangzhou Burning Rock Biotechnology Inc. China) for liver biopsy tissue and peripheral blood were done, including EGFR, HER-2, KRAS, ALK, ROS1, MET, RAT, BRAF, KIT, PDGFRA and so on. Unfortunately, the result revealed no known mutations. The patient has a history of hypertension, type 2 diabetes, multiple arteriosclerosis, the right renal artery stenosis and left subclavian artery stenosis treated with stent implantation one year ago, hyperlipidemia, chronic kidney disease 3a stage and acute cerebrovascular infarction two years ago. On June 24, four major lesions were treated with radiofrequency ablation (RFA), and this treatment was repeated on July 11. The patient had mild adverse effects including fatigue, transient elevated hepatic transaminase (ALT peaked at 280 U/L and AST at 200 U/L) and hypoalbuminemia after RFA. Unfortunately, he developed new lesions seen by enhanced MRI on July 22 (Fig. 1c). Due to rapid progression of his angiosarcoma, the treatment team decided to initiate a combination of targeted therapy and immunotherapy. From July 22, the patient received pazopanib 200 mg daily for 2 days, 400 mg daily for 5 days, then 600 mg daily up to now. He experienced no adverse effects. Due to concern regarding the aggressive behavior of the cancer, on August 1st, pembrolizumab at 100 mg every three weeks was started. Patient experienced no significant adverse effects from this combination. On August 9 the patient received 3 cycles of allogeneic RAK cells therapy. Dose was 3 billion cells daily in 3 consecutive days, given every 4 weeks. The combination of these three therapeutic agents was able to decrease the size and number of the liver masses as showed by MRI on Aug18 (Fig. 1d). Subsequent abdominal enhanced MRI demonstrated stable disease till last image on Oct 26,2017 (Fig. 1e–j). Treatment course timeline is included as Fig. 3. Currently the patient is in stable clinical condition, without any complaints. His Karnofsky performance status (KPS) is 90. Routine laboratory examinations including blood routine, urine routine, blood coagulation, liver and renal function, thyroid gland function, ECG, etc. were all within normal parameters. The patient tolerated pazopanib and pembrolizumab very well. Just after He experienced mild infusion reactions with RAK cell treatment, including transient fever, mild hypertension.

## Discussion and conclusions

PHA is a rare and aggressive solid tumor. It originates from liver vascular endothelial cells and lymphatic vessels cells. An estimated 200 cases are diagnosed annually in the world [3], with a male-to-female ratio of 3:1. This disease has a predisposition to affect those between 60 and 80 years of age [4]. No specific etiologic agent is identified in majority of patients with PHA. Some important risk factors for this disease include the exposure to vinyl chloride, radiocontrast material, the use of androgenic steroid and arsenic, and other chemicals [5].

The symptoms of PHA are nonspecific. Many tumors are found incidentally by diagnostic imaging, others are found as patients present with abdominal pain, anorexia, fatigue, weight loss, fever, and low back pain. Other reported symptoms/findings include jaundice, hemoperitoneum and acute hepatic failure. Primary hepatic angiosarcoma can metastasize to the lungs and hilar lymph nodes [6]. Tumor markers such as AFP, CEA, CA19–9, and CA125 are usually not elevated. This makes PHA difficult to diagnose in its early stage.

Morphologically, PHA appear sole or multiple nodules or a dominant mass, or a mixed pattern. Pathologic diagnosis is golden standard for PHA. The optimal way to obtain tumor tissue is surgery. Percutaneous liver biopsy is considered to be dangerous and unreliable because of the necrosis and hemorrhage inside the tumor. Thus samples taken from these areas may produce false-negative results. Histopathology shows a variety of patterns of vascular channels, dilated sinusoidal or cavernous spaces. CD34, CD31 and factor VIII-related antigen can be positive. Other markers such ERG, Ki-67 and vimentin can be positive [7]. In our case, we did percutaneous core needle liver biopsy two times, first by ultrasound guidance and then by computed tomography (CT).

PHA has high rates of local recurrence and distant metastasis after surgical resection. Prognosis is poor. The median overall survival is 5 months. The causes of death of PHA patients include hepatic failure, disseminated intravascular coagulation, tumor rupture hemorrhagic shock and metastasis. Due to its low incidence, no standard treatment guideline exists for PHA. Some authors reported success with complete resection which can prolong the median survival to 17 months [8]. However, surgical treatment is difficult to carry out in most patients, due to presence of multiple liver metastases or extrahepatic metastasis at the time of diagnosis. Liver transplant is unproved and is not considered to be a viable option due to high recurrence rate. One study reported that the overall median survival of 22 patients who underwent liver transplant was 6 months [9]. Based on our patient’s multiple liver metastases at the time of diagnosis, he was not a good candidate for surgical treatment or liver transplantation. Therefore, he was treated with palliative intent to prolong his survival.

RFA of liver tumors has proven to be an effective way to control local lesions. E. Berber reported that RFA for 18 patients with single liver metastases of sarcoma, local tumor recurrence is about 17% after 24 months of follow up [10]. Therefore, RFA offers an alternative to surgery for inoperable patients. RFA was performed twice in our patient in order to achieve local tumor control. However, the emergence of new lesions rapidly after RFA treatment indicates the aggressive behavior of the cancer, thus making it urgent to start appropriate systemic treatment.

Chemotherapy has been reported to show clinical efficacy in unresectable PHA [8]. Conventional chemotherapeutic agents for treatment of sarcoma include albumin paclitaxel, 5-fluorouracil, carboplatin, doxorubicin, ifosfamide, gemcitabine, dacarbazine, and newer agent trabectedin is approved for soft sarcoma in 2015 [11]. In recent years targeted therapies such as antiangiogenic drugs bevacizumab, sorafenib, and pazopanib showed shown efficacy in treatment of advanced soft tissue sarcoma. The small molecule vascular endothelial growth factor inhibitor pazopanib is a multiple target tyrosine kinase inhibitor, with activity against vascular endothelial growth factors 1, 2, and 3, and platelet derived growth factors. Pazopanib has received approval for the treatment of certain soft tissue sarcomas. In the phase III trial for metastatic soft tissue sarcoma, 372 patients with advanced soft tissue sarcoma whose tumors had progressed despite at least one line of chemotherapy, were randomly assigned either pazopanib or placebo. The difference in progression free survival in the pazopanib arm was statistically significant with a median of 4.6 months for pazopanib compared with 1.6 months for placebo [12]. Given our patient’s more advanced age, we decided chemotherapy would have been too toxic. We instead selected pazopanib as our initial treatment.

In recent years, tumor immunotherapy has become an important therapeutic option for treatment of advanced cancer. Immune checkpoint inhibitors such as anti-PD-1 monoclonal antibodies have shown considerable efficacy in advanced tumors, especially for melanoma, renal cancer, and lung cancer. A retrospective analysis has shown a cohort of patients with relapsed metastatic sarcomas treated with nivolumab, the clinical benefit was observed in 50% of the evaluable patients [13]. Currently, a clinical trial of PD-1 inhibitor nivolumab for sarcoma being conducted in the NCI (NCT02500797). Previous study suggested that targeting the VEGF axis may attenuate tumor-induced immunosuppression, allowing the tumor to become more responsive to immunotherapy when used in combination [14]. It is in this context that Amin et al. designed a phase I clinical trial of nivolumab in combination with sunitinib or pazopanib in metastatic renal clear cell carcinoma (mRCC) NCI(NCT01472081). nivolumab combined with TKI showed encouraging antitumor activity in this study with 53 patients. Objective response rate was higher with combination therapy up to about 50% than seen previously with nivolumab or TKI monotherapy in RCC, with a manageable toxicity profile, although renal and hepatic adverse effects were higher compared to monotherapy. The encouraging results of that study inspired us to consider treating our patient with combination of pazopanib and PD-1 inhibitor. Additionally, if the tumor progressed rapidly, we were afraid the patient’s physical condition may get worse, making him lose the opportunity to treat with pembrolizumab. Therefore, based on pazopanib, we decided to use pembrolizumab as soon as possible. However, considering there is a high incidence of adverse effects with pazopanib, and this case is an elderly man, we worried about his tolerance to combination treatments initially. So, after we determined the patient can tolerable the adverse effects of pazopanib, we started a combination treatment with pembrolizumab.

Moreover, we were concerned that the combination of pazopanib and anti-PD1 monoclonal antibody was insufficient to inhibit the rapid progression of the patient’s primary hepatic angiosarcoma. RAK cells have been reported to be effective in liver, colorectal, lung, pancreatic and other advanced cancers [15, 16]. We hypothesis pazopanib plus PD-1 inhibitor and RAK cells together could bring more benefit in the treatment of our patient. RAK cells is a new kind of cytokine induced killer cells which use the proliferation technique of RetroNectin [17, 18]. One study found that RAK cells are heterogeneous cells in CD3^+^CD8^+^CD56^+^ T cells. These cells appear to secrete more IL-2 and less IL-4 and IL-5, indicating that RetroNectin can potentially enhance the activity of Th1 cells, while suppressing the activity of Th2 cells. This may be a clinically significant advantage for RAK cells, since cancer immunotherapy needs the help of Th1 cells, while the Th2 cells always inhibit antitumor activity of immune system [19]. RAK cells have been shown to grow faster, with a lower ratio of spontaneous apoptosis [17]. Therefore, compared with the traditional technique for cultivating CIK cells, the biggest advantage of RAK cell is the ability to obtain more lymphocytes for the final infusion. In addition, RAK could continue to grow for more days in the patient body, so the therapeutic number of RAK cells needed for cancer patients may be less than that of traditional CIK cells, meanwhile, appearing longer survival time in vitro, possibly leading to a more lasting therapeutic effect after transfusion in vivo [17]. RetroNectin induced autologous and allogeneic cells have some key differences. Even though allogeneic RAK cells have potential for allergic reaction or infections such as viral hepatitis or HIV, it may have potential advantages. In our case, the source of allogeneic RAK cells were from the patient’s children, the donor of allogeneic RAK cells are young and with normal immune ability; besides the anti-tumor effect, allogeneic RAK cells can also stimulate immune responses and repair the low immune surveillance in this patient.

In summary, PHA is a rare disease, our case is characterized by older age, complicated basic disease, rapidly tumor progression, high degree of malignancy. Pazopanib plus PD-1 inhibitor and RAK cells therapy together have worked and control the tumor in a stable situation, side effects are tolerable. From making a definite diagnosis to now, 15 months have passed, the patient feels good, KPS score is 90, follow-up is necessary. The combination of pazopanib, PD-1 inhibitor and RAK cells warrants further exploration as a potential treatment of high advanced PHA.

## Abbreviations

KPS: Karnofsky performance status
NGS: Next generation sequencing
PET/CT: Positron emission tomography/computed tomography
PHA: Primary hepatic angiosarcoma
RAK: Cells RetroNectin-activated killer cells
RFA: Radiofrequency ablation
